# Draft Genome Sequence of the Siderophore-Degrading Soil Bacterium *Mesorhizobium loti* Strain LU

**DOI:** 10.1128/genomeA.00029-18

**Published:** 2018-02-08

**Authors:** Domenic Castignetti, Nathaniel Polley, Catherine Putonti

**Affiliations:** a Department of Biology, Loyola University Chicago, Chicago, Illinois, USA; b Bioinformatics Program, Loyola University Chicago, Chicago, Illinois, USA; c Department of Computer Science, Loyola University Chicago, Chicago, Illinois, USA

## Abstract

Here, we present the draft genome of *Mesorhizobium loti* strain LU, a soil bacterium capable of degrading the trihydroxamate siderophore deferrioxamine B to its constituent monohydroxamic acids. Genome size was 6,399,828 bp, with a GC content of 61.5%. This draft genome consists of 35 scaffolds, with an *N*_50_ of 389,921 bp.

## GENOME ANNOUNCEMENT

The bacterial symbionts, known as the rhizobia, fix atmospheric nitrogen in association with leguminous plants (1). Rhizobia have a significant role in the nitrogen fertility of soils and in agriculture, and the legumes are considered among the most important of agricultural species, second only to the grasses. As crops, the legumes supply the largest source of plant protein in the diets of both humans and livestock (2). In more recent times, the genus *Rhizobium* has been refined and divided into genera such as *Bradyrhizobium*, *Sinorhizobium*, and *Mesorhizobium* (1).

Siderophore degradation has been noted only in a select group of microbes (3). *Mesorhizobium loti* strain LU, isolated from a sample of garden soil in Glenview, IL, USA, is a siderophore-degrading bacterium that synthesizes the constituent monohydroxamic acids from the trihydroxamate siderophore deferrioxamine B and is able to use the siderophore as its sole source of carbon for growth (3). Experimental evidence indicates that the enzyme responsible for deferrioxamine B degradation is most likely a serine peptidase-like enzyme (4).

*M. loti* LU genomic DNA was isolated following growth in enriched medium, such as nutrient broth or tryptic soy broth, using the Wizard genomic DNA purification kit (Promega, Madison, WI) as described by the manufacturer. Genomic DNA concentration was determined using the Qubit fluorimeter. Library preparation for Illumina sequencing was performed by the Loyola University Chicago Genomics Facility; the Nextera XT DNA library preparation kit was used. The library was sequenced on the MiSeq Sequencer (Illumina) using the MiSeq reagent kit v2 (500 cycles). The run produced 1,603,054 paired-end reads in total.

Reads were trimmed using the tool sickle (https://github.com/najoshi/sickle) and assembled using SPAdes (v3.11.1) (5), producing 126 contigs. Coverage was assessed using BBMap (http://sourceforge.net/projects/bbmap/). Contigs with a coverage of less than 1 were removed from further consideration. This resulted in a final set of 35 scaffolds varying in size from 1,001 bp to 707,823 bp (*N*_50_, 389,921 bp) with a coverage of 67×. The genome size was 6,399,828 bp with a GC content of 61.53%. Annotations were produced by the NCBI Prokaryotic Genome Annotation Pipeline using GeneMarkS+ (6). Five rRNA genes, 44 tRNA genes, and 5,989 protein-coding sequences were detected. The annotation pipeline identified 79 pseudogenes within the assembly.

### Accession number(s).

The draft whole-genome project for *Mesorhizobium loti* strain LU has been deposited at DDBJ/EMBL/GenBank under accession number PJRO00000000. Raw sequence reads are deposited at DDBJ/EMBL/GenBank under accession number SRR6382439.

## References

[1] Laranjo M, Alexandre A, Oliveira S 2014 Legume growth-promoting rhizobia: an overview on the *Mesorhizobium* genus. Microbiol Res 169:2–17. doi:10.1016/j.micres.2013.09.012. 24157054

[2] Garg N, Geetanjali N 2007 Symbiotic nitrogen fixation in legume nodules: process and signaling. A review. Agron Sustain Dev 27:59–68. doi:10.1051/agro:2006030.

[3] Pierwola A, Krupinski T, Zalupski P, Chiarelli M, Castignetti D 2004 Degradation pathway and generation of monohydroxamic acids from the trihydroxamate siderophore deferrioxamine B. Appl Environ Microbiol 70:831–836. doi:10.1128/AEM.70.2.831-836.2004. 14766561 PMC348822

[4] Zaya N, Roginsky A, Williams J, Castignetti D 1998 Evidence that a deferrioxamine B degrading enzyme is a serine protease. Can J Microbiol 44:521–527. doi:10.1139/w98-031. 9734303

[5] Bankevich A, Nurk S, Antipov D, Gurevich AA, Dvorkin M, Kulikov AS, Lesin VM, Nikolenko SI, Pham S, Prjibelski AD, Pyshkin AV, Sirotkin AV, Vyahhi N, Tesler G, Alekseyev MA, Pevzner PA 2012 SPAdes: a new genome assembly algorithm and its applications to single-cell sequencing. J Comput Biol 19:455–477. doi:10.1089/cmb.2012.0021. 22506599 PMC3342519

[6] Tatusova T, DiCuccio M, Badretdin A, Chetvernin V, Nawrocki EP, Zaslavsky L, Lomsadze A, Pruitt KD, Borodovsky M, Ostell J 2016 NCBI Prokaryotic Genome Annotation Pipeline. Nucleic Acids Res 44:6614–6624. doi:10.1093/nar/gkw569. 27342282 PMC5001611

